# Psychometric properties of the Reintegration to Normal Living Index for sepsis survivors

**DOI:** 10.1007/s11136-023-03403-3

**Published:** 2023-03-30

**Authors:** Kathleen Streich, Christiane S. Hartog, Carolin Fleischmann-Struzek, Norman Rose, Anna Bichmann, Miriam Kesselmeier, Fridtjof Schiefenhövel, Malte Schmieding, Sebastian Born

**Affiliations:** 1 Department of Anesthesiology and Intensive Care, Charité-Universitätsmedizin Berlin, Freie Universität Berlin and Humboldt-Universität Zu Berlin, Berlin, Germany; 2 Klinik Bavaria, Kreischa, Germany; 3 Institute of Infectious Diseases and Infection Control, Jena University Hospital, Stoystraße 3, 07743 Jena, Germany; 4 Center for Sepsis Control and Care, Jena University Hospital, Jena, Germany; 5 Institute of Medical Statistics, Computer and Data Sciences, Jena University Hospital, Jena, Germany

**Keywords:** Sepsis, Reintegration to Normal Living Index, Psychometric properties, Factor analysis, Rehabilitation, Activities of daily living

## Abstract

**Purpose:**

Return to a normal state of living is a key patient-relevant outcome for sepsis survivors. The Reintegration to Normal Living Index (RNLI) assesses self-perceived participation in patients with chronic disease, but its psychometric properties have been analyzed neither for patients after sepsis nor in a German patient cohort. This study aims to analyze the psychometric properties of the German version of the RNLI in sepsis survivors.

**Methods:**

In a prospective multicenter survey study, 287 sepsis survivors were interviewed 6 and 12 months after hospital discharge. Multiple-group categorical confirmatory factor analyses with three competing models were used to explore the factor structure of the RNLI. Concurrent validity was evaluated in relation to the EQ-5D-3L and the Barthel Index of Activities of Daily Living (ADL).

**Results:**

Regarding structural validity, all models showed an acceptable model fit. Because of high correlation between the latent variables in the two-factor models (up to *r* = 0.969) and for reason of parsimony, we opted for the common factor model to analyze the concurrent validity. Our analyses showed moderate positive correlations between RNLI score and ADL score (*r* ≥ 0.630), EQ-5D-3L visual analogue scale (*r* ≥ 0.656) and EQ-5D-3L utility score (*r* ≥ 0.548). The reliability assessed by McDonald’s Omega was 0.94.

**Conclusion:**

We found convincing evidence for good reliability, structural and concurrent validity of the RNLI in German sepsis survivors. We propose to use the RNLI in addition to generic health-related quality of life measures to assess the reintegration to normal living after sepsis.

**Supplementary Information:**

The online version contains supplementary material available at 10.1007/s11136-023-03403-3.

## Introduction

Sepsis is a dysregulated severe immune response to infection [1]. In 2015, 320,000 patients with sepsis received hospital treatment in Germany, of which 76% survived their hospital admission [2]. Sepsis survivors often suffer from long-lasting and debilitating sequelae [3], including chronic respiratory or renal impairment, critical illness polyneuropathy/myopathy, fatigue, post-traumatic stress disorder, anxiety and depression [4–6]. A recent cohort study among 116,507 survivors of hospital-treated sepsis in Germany found that 74% of survivors suffered from new medical, cognitive, or psychological diagnoses in the first year after sepsis and nearly one-third of survivors at risk were newly dependent on nursing care [7]. Hence, sepsis is considered a life-changing disease with its sequelae leading to long-term impairments in patients’ health-related quality of life (HRQL) [8]. In consequence, the World Health Organization (WHO) highlighted the need for further research to improve the understanding of the effects of sepsis on the patients’ quality of life, and to identify effective interventions to improve long-term outcomes, including the HRQL [9]. The HRQL in sepsis survivors is commonly assessed by the Short Form 36 Health Survey (SF-36) or the EuroQoL-5D (EQ-5D) [10]. However, sepsis survivors themselves describe many aspects that are not captured by these generic HRQL instruments [11]. There has been a predominant highlight on the importance to the return to a normal state of living after hospital discharge [11]. According to Wood-Dauphinée [12], return to a state of normal living does not only imply the return to a pre-illness state. Moreover, it comprises the acceptance of and the adjustment to changed living conditions.

Return to normal living can be measured by the Reintegration to Normal Living Index (RNLI), an instrument that was developed to quantify the recovery of global function after trauma or serious illness [12, 13]. The RNLI has been validated for patients after stroke, spinal cord injury or in older adults, but not for sepsis survivors and/or within a German patient cohort [14]. Previous studies examining the psychometric properties of the RNLI for different patient cohorts showed good internal consistency (Cronbach’s alpha: 0.73-0.97), test-retest reliability and discriminant validity [14]. The factor structure though remains debated. Depending on the cohort, a common factor solution as well as different two-factor solutions have been reported [12, 15–21].

However, psychometric properties of measurement instruments can vary across populations (e.g., violation of the assumptions of measurement invariance) [22, 23]. Furthermore, the translations of a questionnaire into another language can unintentionally affect the factor structure, reliability, validity, and other properties of the instrument [24]. Therefore, the present study aims to provide evidence for two aspects of construct validity: (1) the underlying factor structure of the German version of the RNLI (i.e., structural validity) and (2) the concurrent validity based on correlations with other clinical instruments used to measure aspects of global functioning and HRQL in a German population of sepsis survivors.

## Methods

### Setting and participants

The data collection was part of a prospective longitudinal survey study (SEPFROK study, DRKS preregistration: DRKS00016340) conducted between February 2019 and September 2020. Patients were recruited from the intensive care units (ICU) of two university hospitals (Charité -Universitätsmedizin Berlin, Jena University Hospital). Furthermore, the Mid-German Sepsis Cohort (MSC) served as recruiting platform for this survey study. The MSC included patients with sepsis from ICUs in the university hospitals of Jena, Leipzig and Halle, and non-academic hospitals in Erfurt and Bad Berka [25], who were invited to participate in this survey study during their MSC follow-up assessments.

Potential participants were identified prospectively by documented International Classification of Diseases, 10th Revision, German Modification (ICD-10-GM) codes for sepsis and septic shock (Berlin) or daily screening for clinical sepsis criteria (Jena, MSC) [25] of all ICU patients. We included patients ≥ 18 years of age, who were able to give consent or had a relative as legal representative to consent on their behalf to participate in this survey study according to the Institutional Review Boards (IRB) requirements. We excluded patients not fluent in the German language and patients with professional legal representatives without a personal relationship. All interviews were conducted at 6 and 12 months after discharge from the ICU (± 6 weeks) by trained investigators. Patients who did not participate in the first interview could participate in the second interview. Patients who were not able to participate in the survey independently could answer the questions with support of an informal caregiver or through that person on their behalf. Ethical approval for this study was obtained by the IRB of the Friedrich-Schiller University Jena (2018–1223-Bef) and the Charité-Universitätsmedizin Berlin (EA4/060/19).

### Measures

The interview was previously developed to assess the utilization and satisfaction with rehabilitation and aftercare after sepsis in a longitudinal cohort of sepsis survivors and included questions on patient demographics, reintegration to normal living measured by the RNLI, the Barthel Index for Activities of Daily Living (ADL) and the EQ-5D-3L as measure of HRQL.

#### RNLI

The RNLI was originally developed to quantify the degree of reintegration after trauma or incapacitating disease including stroke [12]. The instrument consists of eleven declarative statements on physical, functional, psychological and social aspects of life. The participants can respond on a four-point Likert scale (1 = disagree, 2 = somewhat disagree, 3 = somewhat agree, 4 = agree) to indicate how much a statement applies to their current living situation. Higher scores represent higher level of reintegration. In previous research, different response formats have been used, including a ten-point visual analog scale (VAS) [12], a ten-point Likert scale [16], a three-point Likert scale [18; 26], and a four-point Likert scale [27]. We decided to use the four-point Likert scale, because of its simplicity and yet sufficient discrimination of the different levels of agreement [17]. A German version of the RNLI was translated from the English original version of Wood-Dauphinee et al. [12] (Supplementary Table 1). The translation into German followed standardized procedures with forward and backward translation by a licensed translator/native speaker.

#### ADL

The ADL assesses patients’ functional independency. It was developed to monitor improvement in the rehabilitation of the chronically ill [28]. The ADL includes ten items with two to three answer options (unable, needs help, independent), which rate a person’s ability to function independently regarding feeding, bathing, grooming, dressing, patients’ bowel and bladder control, toilet use and transfers (e.g., bed to chair). A sum score is calculated, with higher scores denoting a higher degree of independence.

#### EQ-5D-3L

The EQ-5D-3L, developed by the EuroQol group, is a standardized generic instrument for describing and evaluating HRQL aspects [29]. It includes a visual analogue scale (VAS) to rate the general health status from 0 to 100, in which 100 denotes the best possible health. Furthermore, it assesses five dimensions of HRQL: mobility, self-care, usual activities, pain/discomfort and anxiety/depression with three possible degrees of impairment (no problems, some problems, severe problems) [29]. The answers result in a five-digit score, which can be converted into a single utility index score with the R package eq5d by Fraser Morton and Jagtar Singh Nijjar using a country specific value set [30].

### Analyses

The collected interview data combines observations from different time points (6 and 12 months after ICU discharge) and different reporter types (patients, patients with support of informal caregivers, or informal caregivers alone). To achieve a sufficient sample size per group, we decided to include only two reporter types (patients and informal caregiver with/without patient). Group 1 includes all interviews in which the patients participated independently 6 months after discharge from ICU. Group 2 contains all interviews that were conducted with patients supported or performed by an informal caregiver (e.g., husband, wife) at 6 months after ICU discharge.

Descriptive statistics were calculated for demographic characteristics and clinical features for the two subgroups. We report means and standard deviations for metric data, and frequencies and percentages of the total amount for nominal data. Categorical Confirmatory Factor Analysis (CCFA) [31] was used to examine the underlying factor structure of the German version of the RNLI. We selected competing models for the RNLI based on a literature review (Table 1). Model 1 was a single factor solution, established by Mothabeng et al. for patients with spinal cord injuries [19]. Merz et al. confirmed this single factor structure in a stroke population [20]. Model 2 was a two-factor solution postulated by Wood-Dauphinee et al. [12, 13] and confirmed by Hitzig et al. [18] and Daneski et al. [15], defining items one to eight as daily functioning and items nine to eleven as perception of self. Model 3 is an alternative two-factor solution proposed by Miller et al. [17], in which items one to seven form the factor daily functioning and items eight to eleven the factor personal integration.

**Table 1:** Structure of existing models for the reintegration to normal living index

Model	Structure	Assignment	Meaning	Studies
1	Common factor	Item 1–11	Reintegration to Normal Living	Mothabeng et al. [19]
2	Two factors	Item 1–8; Item 9–11	Daily Functioning; Perception of Self	Wood-Dauphinee et al. [12]; Hitzig et al. [18]; Daneski et al. [15]
3	Two factors	Item 1–7; Item 8–11	Daily Functioning; Personal Integration	Miller et al. [17]

Because analyzing the factor structure of the RNLI without considering the subgroups of the study population would implicitly assume measurement invariance across the reporter types, we opted for a multiple-group structural equation model (SEM), with the assumption of strong measurement invariance [22, 32]. Hence, we specified the model including equality constraints for the factor loadings and thresholds across the groups. The mean of the latent variable in the first group was fixed to zero for purpose of model identification. All analyses were carried out using the R package lavaan [33]. According to the response format (e.g., Likert scale), the items were considered as ordinal indicators and model parameters were estimated with the mean and variance adjusted weighted least square (WLSMV) estimator [34] based on the polychoric correlations of the items. Following the recommendation of Beauducel and Wittmann [35], we used multiple criteria (e.g., model fit indices) to evaluate the fit of the different models: (1) Root Mean Square Error of Approximation (RMSEA), with RMSEA < 0.08 indicating reasonable and RMSEA < 0.05 indicating good fit; (2) Comparative Fit Index (CFI); (3) Tucker Lewis Index (TLI), in which values equal or greater than 0.95 indicate good fit, and (4) Standardized Root Mean Residual (SRMR), in which values less than 0.05 indicate good fit [35–38].

To examine concurrent validity, we extended the selected the measurement model of the RNLI by incorporating the ADL and EQ-5D-3L VAS scores as well as the EQ-5D-3L utility score as further clinical test scores (hereinafter referred to as Model 4). Correlations between the latent RNLI variable and the test scores were freely estimated. Because the item content of all three instruments refers to aspects of daily functioning (e.g., self-care, usual activities, mobility), but also to unique aspects such as subjective pain, anxiety and depression (EQ-5D-3L) or social aspects of life (RNLI), we expected substantial correlations between these factors. According to Hinkle, Jurs and Wiersma [39] this corresponds to a moderate to strong relationship. For assessing the reliability of the RNLI test score, we used McDonald’s Omega [40], which has proven superior to Cronbach’s α in cases of unequal factor loadings and missing item responses [41]. All analyses were performed in R Version 4.2.2 [42]. We applied a significance level of α = 0.05. For the correlations, we provided Wald confidence intervals. If confidence limits were outside of the parameter space of correlations, we set it to the boundary values of −1 or 1.

## Results

### Participants

A total of 287 sepsis survivors participated in this prospective multicenter survey study, of which two did not provide answers on the RNLI. Supplementary Fig. 1 shows the patient flow including excluded and included patients. In total, 400 interviews were conducted, with 226 used for the analysis of the psychometric properties of the RNLI. The interviews analyzed were conducted with patients (67.3%) and patients supported by informal caregivers or care givers alone (32.7%). Patient characteristics are summarized for each of the two groups in Table 2. Mean patient age was 62.8 ± 13.9 (standard deviation, SD) years in Group 1 and 67.5 ± 12.0 (SD) years in Group 2. The proportion of female participants slightly differed between the groups (Group 1: 34.2%, Group 2: 32.4%). Furthermore, patients who needed support to conduct the interview (Group 2) stayed in the hospital 35% longer and were four times as likely dependent on nursing care prior to sepsis than patients who independently conducted the interviews (Group 1). Regarding employment state prior sepsis, the patients of Group 1 were three times more likely to work full- or part-time than patients of Group 2. Further patient characteristics are displayed in Table 2.

**Table 2:** Characteristics for the different groups of the study population

Study population (*n* = 226)	Group 1 (*n* = 152)	Group 2 (*n* = 74)
*Patient characteristics*		
Female gender, *n* (%)	52 (34.2)	24 (32.4)
Age in years, mean (SD)	62.8 (13.9)	67.5 (12.0)
Education		
≤ 10 years of school education, *n* (%)	107 (72.3)	56 (81.2)
> 10 years of school education, *n* (%)	40 (27.0)	13 (18.8)
Others, *n* (%)	1 (0.7)	0
N. a., *n* (%)	4 (2,6)	5 (6.8)
Working (full-/part-time)		
Prior to sepsis, *n* (%)	65 (43.3)	11 (14.9)
At time of interview, *n* (%)	25 (16.7)	1 (1.4)
Nursing care dependency		
Prior to sepsis, *n* (%)	11 (7.3)	23 (31.1)
At time of interview, *n* (%)	52 (34.7)	52 (73.2)
*Clinical features*		
Sepsis origin		
Community-acquired infection, *n* (%)	48 (31.6)	25 (33.8)
Hospital-acquired infection, *n* (%)	92 (60.5)	46 (62.2)
Unknown, *n* (%)	12 (7.9)	3 (4.1)
Length of hospital stay in days, mean (SD)	38.5 (29.0)	51.8 (58.8)
Maximum SOFA Score, mean (SD)	10.8 (3.7)	11.6 (3.7)

*Note:* Group 1 = patients interviewed 6 months after sepsis, Group 2 = informal caregiver or both interviewed 6 months after sepsis. n.a. = not answered. Absolute and percentage frequencies (*n*, %) or mean with standard deviation (SD) are provided

### Categorical confirmatory factor analysis

Table 3 reports the model fit indicators for the examined models 1–3. The model fit indices CFI, TLI, RMSEA and SRMR indicated acceptable fit of all three models. Item thresholds and item factor loadings for the different models are reported in Table 4, Supplementary Table 4a and b. Absolute and relative response frequencies are shown in Supplementary Table 3. In all models, the item factor loadings for all items were positive and differed significantly from zero (*p* < 0.001). A considerably lower factor loading was found for item 10 (“In general, I am comfortable with myself when I am in the company of others.”) compared to the other factor loadings. The correlation between the factors “Daily Functioning” and “Perception of Self” in Model 2 for the two subgroups were *r* = 0.857 and *r* = 0.969. For Model 3, the correlation between the factors “Daily Functioning” and “Personal Integration” were *r* = 0.898 and *r* = 0.933. All correlations including Wald confidence intervals are reported in Supplementary Table 5.

**Table 3:** Model fit indices of the different models for the RNLI

Model	n	${\chi }^{2}$	df	p-value (${\chi }^{2}$)	CFI	TLI	RMSEA	90% CI for RMSEA	SRMR
1	226	182.335	119	< 0.001	0.984	0.985	0.069	[0.048, 0.088]	0.070
2	226	164.115	115	0.002	0.988	0.988	0.062	[0.039, 0.082]	0.064
3	226	160.345	115	0.003	0.989	0.989	0.059	[0.035, 0.080]	0.062
4	226	257.464	179	< 0.001	0.977	0.977	0.063	[0.045, 0.079]	0.069

*Note:* RNLI = Return to Normal Living Index. Model 1: Common Factor. Model 2: Two Factors (Daily Functioning, Perception of Self). Model 3: Two Factors (Daily Functioning, Personal Integration). Model 4: Extended Model 1 with Covariates (EQ5D-3L-VAS, EQ5D-3L Utility Score, ADL)

**Table 4:** Thresholds of the items and factor loadings of model 1 for the RNLI

Item	Thresholds			Factor loadings
	Disagree/somewhat disagree	Somewhat disagree/somewhat agree	Somewhat agree/agree	Common factor (Reintegration to Normal Living)
1	−1.552	−1.132	−0.632	0.751
2	−1.308	−0.799	−0.219	0.892
3	−0.722	−0.349	−0.040	0.860
4	−1.903	−1.235	−0.593	0.794
5	−1.101	−0.630	−0.054	0.863
6	−0.888	−0.350	0.287	0.882
7	−1.190	−0.551	0.035	0.825
8	−1.453	−0.999	−0.329	0.868
9	−1.786	−1.322	−0.707	0.909
10	−2.082	−1.167	−0.483	0.511
11	−1.678	−0.992	-0.117	0.826

*Note:* RNLI = Return to Normal Living Index. Factor structure of Model 1 corresponds to the common factor structure proposed by Mothabeng et al. ( [19]. All factor loadings are significant with *p* < 0.001

Because of the high correlation between the factors in the two-factor models and for the reason of parsimony [43], i.e., the scientific principle that in case of two models under identical conditions, the simpler one should be preferred, we opted for Model 1 and used this model to analyze the concurrent validity. The means of the common factor “Reintegration to Normal Living” (RNL) in Model 1 significantly differed between the reporter types. Patients who completed the interview without support had a mean of zero in Group 1 (reference category). In contrast, the mean of the common factor among patients who needed support to complete the interview (Group 2) was significantly lower (−0.876, 95% CI [−1.207, −0.545]). For the common factor model, McDonald’s Omega reliability estimate was 0.94, 95% CI [0.92, 0.95].

### Concurrent validity

The extended model (Model 4) with the correlated tests scores (e.g., ADL, EQ-5D-3L VAS scores, and EQ-5D-3L Utility Score) showed an acceptable model fit (CFI 0.976, TLI 0.976, RMSEA 0.064, SRMR 0.069). The correlations between the latent RNL variable and the scores of the additional clinical measures (see Table 5) were similar across the two groups, and ranged from *r* = 0.548 and *r* = 0.656 in Group 1, and from *r* = 0.571 to *r* = 0.756 in Group 2. All correlations differed significantly from zero (*p* < 0.001). As expected, the Common Factor RNL showed moderate to strong positive correlation with the other clinical instruments, indicating good concurrent validity.

**Table 5:** Correlations with 95% confidence intervals between the common factor underlying the RNLI and other clinical instruments for the different groups

Group	Factor	RNLI	EQ-5D-3L VAS	EQ-5D-3L utility score
Group 1	EQ-5D-3L VAS	0.656 [0.556, 0.755]		
(*n* = 152)	EQ-5D-3L Utility Score	0.568 [0.448, 0.688]	0.548 [0.446, 0.649]	
	ADL	0.630 [0.529, 0.731]	0.631 [0.528, 0.734]	0.634 [0.539, 0.728]
Group 2	EQ-5D-3L VAS	0.656 [0.505, 0.806]		
(*n* = 74)	EQ-5D-3L Utility Score	0.641 [0.473, 0.810]	0.571 [0.389, 0.754]	
	ADL	0.710 [0.561, 0.859]	0.682 [0.531, 0.834]	0.756 [0.628, 0.885]

*Note:* Group 1 = patients interviewed 6 months after sepsis, Group 2 = informal caregiver or both interviewed 6 months after sepsis. RNLI = Return to Normal Living Index, VAS = Visual Analog Scale, ADL = Barthel Index of Activities of Daily Living. All correlations are significant with *p* < 0.001

## Discussion

The psychometric modeling of the RNLI data suggests that the RNLI serves as reliable measure to assess the reintegration into normal living in German sepsis survivors. All three competing models demonstrated acceptable fit. Given the high correlation between latent variables in the two-factor models and considering the criterion of parsimony [43], the common factor model is the model of choice in the population of German sepsis survivors. Furthermore, the results support the assumption of measurement invariance across reporter types. We found significant difference in the latent variable RNL between groups of patients with and without support. As expected, we found moderate positive correlations of the RNLI with the EQ-5D-3L and the ADL, indicating good concurrent validity.

Our results that favor the common factor solution are in line with previous studies, e.g., by Mothabeng et al. [19]. Although a two-factor solution is theoretically possible based on the item content, the high correlation between the two factors speaks for the common factor solution. Furthermore, it was apparent that in all models the item 10 (“In general, I am comfortable with myself when I am in the company of others.”) had a lower factor loading compared to the other items. After examining the wording of the item, we assume that this question is far too general for assessing reintegration after a severe illness. Hence, the response behavior to item 10 may strongly be affected by the individuals’ personality traits, such as extraversion. This ambiguity may explain the lower item factor loading. There are several possibilities to address this issue. One way is a revision of item 10 regarding the peculiarities of social living of sepsis survivors. This may improve the model fit and lead to a higher factor loading of item 10 as well as increased reliability. Another possibility could be an exclusion of item 10 from the instrument, given that the concept of reintegration to normal living is sufficiently represented by the remaining items. Regardless of the chosen solution, the psychometric properties of a modified version of the RNLI would have to be reanalyzed in further studies.

Using the RNLI in clinical studies of sepsis survivorship in addition to a generic HRQL assessment like the EQ-5D-3L may have several advantages. The questionnaire is short and does not take more than three to five minutes to answer. If a common factor model is used, the evaluation is simplified because only one score needs to be calculated and interpreted. Unlike instruments for measuring generic HRQL that focus mainly on daily functioning, or psychological and social aspects, the RNLI assesses to what extent the survivor of severe illnesses resume relatively normal living patterns [12]. For sepsis survivors, this is of overarching importance. Thus, the RNLI can also be considered as an addition to the core outcome set for clinical research in this population.

Our study has several strengths, including the prospective design and the inclusion of patients from six different hospitals including two non-academic institutions. Patients were treated on multidisciplinary ICUs including, e.g., general surgical, neurosurgical, cardiological, medical ICUs and identified based on a complete daily screening, which increases generalizability.

There are also limitations to be considered. The requirement of obtaining written informed consent prior to participation in the study may have deterred some potential participants and thus may have led to a systematic drop out, which limits generalizability. Similarly, our sample only comprises ICU-treated sepsis survivors. Non-ICU-treated patients remained unconsidered reducing the generalizability of our findings to the overall cohort of sepsis survivors as well. The multiple-group model was used to address the heterogeneity of our sample due to the individual differences in the need for support when answering the RNLI. However, the sample sizes within groups are quite small especially for CCFA models. There might be other sources of systematic differences in the underlying construct as well as in the response behavior to the RNLI. Such sources of variation could be addressed and quantified with sufficiently large samples using more complex CCFA or with variance component models of the generalizability theory [44]. Furthermore, CCFA rest upon the testable assumption of underlying multivariate normality [45]. Significant violations of the normality assumption are found in Group 2 (*p* = 0.049) but not in Group 1 (*p* = 0.153). Despite robustness of CCFA against moderate violations of the normality assumptions, biased parameter estimates due to non-normality cannot be ruled out [45, 46].

## Conclusions

There is empirical evidence for good reliability, structural and concurrent validity of the German version of the RNLI in the German population of sepsis survivors. Thus, it could be used in addition to generic HRQL measures to assess the state of a reintegration into normal living among sepsis survivors. Future research should investigate the applicability of the RNLI in survivors of sepsis and critical care, including reliability and responsiveness in non-ICU-treated sepsis survivors.

## Supplementary Information

Below is the link to the electronic supplementary material.

Supplementary file1 (DOCX 61 KB)

## Data Availability

The raw data supporting the conclusions of this article will be made available by the authors, without undue reservation.
