# Supplementary material for: Psychometric properties of the Reintegration to Normal Living Index for sepsis survivors
Source: Qual Life Res. 2023 Mar 30;32(8):2415–23. doi: 10.1007/s11136-023-03403-3 (PMC10328882; doi:10.1007/s11136-023-03403-3)
Supplement: Supplementary file 1 — Supplementary file1 (DOCX 61 KB) [file 11136_2023_3403_MOESM1_ESM.docx]

**Supplement**

**Supplementary Table 1**

*German Version of the RNLI*

| **Item** |  |  |
| --- | --- | --- |
| 1 |  | Ich kann mich in meiner häuslichen Umgebung meinen Bedürfnissen entsprechend bewegen. |
| 2 |  | Ich kann mich in meinem sozialen Umfeld meinen Bedürfnissen entsprechend bewegen. |
| 3 |  | Ich kann Ausflüge außerhalb meines Wohnortes machen, falls ich das möchte. |
| 4 |  | Ich bin zufrieden damit, wie meine Alltagsbedürfnisse erfüllt werden (Ankleiden, Nahrungsaufnahme, Toilettengänge, Baden/Duschen). |
| 5 |  | Ich gehe an den meisten Tagen einer Tätigkeit nach, die notwendig oder mir wichtig ist. |
| 6 |  | Ich bin in der Lage, den von mir gewünschten Freizeitaktivitäten nachzugehen (Hobbys, handwerkliche Tätigkeiten, Sport, Lesen, Fernsehen, Spielen, Computer, etc.). |
| 7 |  | Ich nehme im nötigen oder von mir gewünschten Umfang an sozialen Aktivitäten mit Familie, Freunden und/ oder Kollegen und Geschäftsfreunden teil. |
| 8 |  | Ich übernehme innerhalb meiner Familie eine Rolle, die meinen Bedürfnissen und denen meiner Familienmitglieder entspricht. |
| 9 |  | Im Allgemeinen bin ich mit meinen persönlichen Beziehungen zu anderen Menschen zufrieden. |
| 10 |  | Im Allgemeinen fühle ich mich in Gesellschaft anderer Menschen wohl. |
| 11 |  | Ich glaube, dass ich den Herausforderungen meines Lebens gewachsen bin. |
| *Note*. RNLI = Reintegration to Normal Living Index, English version from Wood-Dauphinee et al. (1988) | | |

**Supplementary Table 2**

*Sepsis Criteria*

| Item | Definition |
| --- | --- |
| Sepsis | Presence of infection (I.) AND at least one acute organ dysfunction (II.) |
| Septic shock | Presence of infection (I.) AND hypotension despite sufficient volume substitution (without other cardiogenic or hypovolemic causes) (III.) |
| with |  |
| 1. Infection | Microbiologically proven or clinically suspected |
| 1. Organ dysfunction | - Acute encephalopathy (impaired vigilance, disorientation, agitation, delirium) - Relative or absolute thrombocytopenia (decrease of >30% within 24 hours or number of platelets ≤100.000/mm^3^ (without other cause)) - Arterial hypoxemia (PaO_2_ ≤10 kPa (≤75 mmHg) under ambient air or PaO_2_/FiO_2_-ratio ≤33 kPa (≤250 mmHg) with oxygenation) - Renal dysfunction (diuresis ≤0,5 ml/kg/h for at least 2 hours despite sufficient volume substitution and/or increase of serum creatinine > 2× above locally common reference) - Metabolic acidosis (base excess ≤-5 mmol/l or blood lactate > 1,5× above locally common reference) |
| 1. Hypotension | - Systolic arterial blood pressure ≤90 mmHg for at least 1 hour, and mean arterial blood pressure ≤65 mmHg, respectively   OR   - Need of vasopressor support to raise systolic arterial blood pressure ≥90 mmHg and mean arterial blood pressure ≥65 mmHg, respectively |

**Supplementary Table 3**

*Absolute and Relative Response Frequencies with 95% Confidence Interval for the Items of the RNLI*

| **Item** |  | **Absolute Response Frequencies** | | | | |  | **Relative Response Frequencies [95% CI]** | | | | |  |
| --- | --- | --- | --- | --- | --- | --- | --- | --- | --- | --- | --- | --- | --- |
|  |  | **1** | **2** | **3** | **4** | **n.a.** |  | **1** | **2** | **3** | **4** | **n.a.** |  |
| 1 |  | 32 | 18 | 33 | 142 | 1 |  | 0.142 [0.084, 0.207] | 0.080 [0.022, 0.145] | 0.146 [0.088, 0.211] | 0.628 [0.571, 0.694] | 0.004 [0.000, 0.070] |  |
| 2 |  | 45 | 29 | 42 | 109 | 1 |  | 0.199 [0.133, 0.266] | 0.128 [0.062, 0.195] | 0.186 [0.119, 0.253] | 0.482 [0.416, 0.549] | 0.004 [0.000, 0.072] |  |
| 3 |  | 79 | 27 | 24 | 94 | 2 |  | 0.350 [0.283, 0.420] | 0.119 [0.053, 0.190] | 0.106 [0.040, 0.177] | 0.416 [0.350, 0.487] | 0.009 [0.000, 0.080] |  |
| 4 |  | 19 | 25 | 38 | 137 | 7 |  | 0.084 [0.022, 0.146] | 0.111 [0.049, 0.173] | 0.168 [0.106, 0.230] | 0.606 [0.544, 0.668] | 0.031 [0.000, 0.093] |  |
| 5 |  | 53 | 31 | 44 | 96 | 2 |  | 0.235 [0.168, 0.304] | 0.137 [0.071, 0.207] | 0.195 [0.128, 0.264] | 0.425 [0.358, 0.494] | 0.009 [0.000, 0.078] |  |
| 6 |  | 66 | 39 | 49 | 69 | 3 |  | 0.292 [0.226, 0.363] | 0.173 [0.106, 0.243] | 0.217 [0.150, 0.288] | 0.305 [0.239, 0.376] | 0.013 [0.000, 0.084] |  |
| 7 |  | 49 | 41 | 45 | 90 | 1 |  | 0.217 [0.150, 0.287] | 0.181 [0.115, 0.251] | 0.199 [0.133, 0.269] | 0.398 [0.332, 0.468] | 0.004 [0.000, 0.074] |  |
| 8 |  | 39 | 21 | 46 | 115 | 5 |  | 0.173 [0.111, 0.243] | 0.093 [0.031, 0.163] | 0.204 [0.142, 0.274] | 0.509 [0.447, 0.579] | 0.022 [0.000, 0.092] |  |
| 9 |  | 21 | 18 | 38 | 147 | 2 |  | 0.093 [0.035, 0.156] | 0.080 [0.022, 0.143] | 0.168 [0.111, 0.231] | 0.650 [0.593, 0.714] | 0.009 [0.000, 0.072] |  |
| 10 |  | 15 | 28 | 45 | 135 | 3 |  | 0.066 [0.004, 0.131] | 0.124 [0.062, 0.189] | 0.199 [0.137, 0.264] | 0.597 [0.535, 0.662] | 0.013 [0.000, 0.078] |  |
| 11 |  | 25 | 30 | 63 | 101 | 7 |  | 0.111 [0.044, 0.179] | 0.133 [0.066, 0.201] | 0.279 [0.212, 0.347] | 0.447 [0.381, 0.516] | 0.031 [0.000, 0.100] |  |
| *Note*. CI = confidence interval. RNLI = Return to Normal Living Index (answers on a 4-point Likert scale). n.a. = not answered. | | | | | | | | | | | | | |

**Supplementary Table 4a**

*Thresholds of the Items and Factor Loadings of Model 2 for the RNLI*

| **Item** |  | **Thresholds** | | |  | **Factor Loadings** | |
| --- | --- | --- | --- | --- | --- | --- | --- |
|  |  | Disagree/ Somewhat Disagree | Somewhat Disagree/ Somewhat Agree | Somewhat Agree/ Agree |  | Factor 1 Daily Functioning | Factor 2 Perception of Self |
| 1 |  | -1.564 | -1.143 | -0.641 |  | 0.781 |  |
| 2 |  | -1.322 | -0.811 | -0.232 |  | 0.874 |  |
| 3 |  | -0.733 | -0.359 | -0.049 |  | 0.880 |  |
| 4 |  | -1.914 | -1.246 | -0.605 |  | 0.767 |  |
| 5 |  | -1.114 | -0.643 | -0.066 |  | 0.795 |  |
| 6 |  | -0.901 | -0.362 | 0.277 |  | 0.798 |  |
| 7 |  | -1.203 | -0.562 | 0.025 |  | 0.852 |  |
| 8 |  | -1.470 | -1.013 | -0.341 |  | 0.903 |  |
| 9 |  | -1.795 | -1.281 | -0.637 |  |  | 0.865 |
| 10 |  | -2.158 | -1.147 | -0.459 |  |  | 0.611 |
| 11 |  | -1.662 | -0.934 | -0.029 |  |  | 0.902 |
| *Note:* RNLI = Return to Normal Living Index. Factor structure of model 2 corresponds to the structure proposed by Wood-Dauphinee et al. (1988), Hitzig et al. (2012, and Daneski et al. (2003). All factor loadings are significant with p < 0.001. | | | | | | | |

**Supplementary Table 4b**

*Thresholds of the Items and Factor Loadings of Model 3 for the RNLI*

| **Item** |  | **Thresholds** | | |  | **Factor Loadings** | |
| --- | --- | --- | --- | --- | --- | --- | --- |
|  |  | Disagree/ Somewhat Disagree | Somewhat Disagree/ Somewhat Agree | Somewhat Agree/ Agree |  | Factor 1 Daily Functioning | Factor 2 Personal Integration |
| 1 |  | -1.570 | -1.147 | -0.645 |  | 0.788 |  |
| 2 |  | -1.327 | -0.816 | -0.235 |  | 0.880 |  |
| 3 |  | -0.737 | -0.362 | -0.052 |  | 0.883 |  |
| 4 |  | -1.914 | -1.249 | -0.609 |  | 0.771 |  |
| 5 |  | -1.119 | -0.647 | -0.069 |  | 0.801 |  |
| 6 |  | -0.905 | -0.365 | 0.274 |  | 0.803 |  |
| 7 |  | -1.207 | -0.565 | 0.022 |  | 0.857 |  |
| 8 |  | -1.433 | -0.957 | -0.281 |  |  | 0.952 |
| 9 |  | -1.798 | -1.296 | -0.658 |  |  | 0.815 |
| 10 |  | -2.147 | -1.155 | -0.467 |  |  | 0.588 |
| 11 |  | -1.664 | -0.950 | -0.054 |  |  | 0.832 |
| *Note:* RNLI = Return to Normal Living Index. Factor structure of model 3 corresponds to the structure proposed by Miller et al. (2011). All factor loadings are significant with p < 0.001. | | | | | | | |

**Supplementary Table 4c**

*Thresholds of the Items and Factor Loadings of Model 4 (Extended Model 1 with Covariates)*

| **Item** |  | **Thresholds** | | |  | **Factor Loadings** |
| --- | --- | --- | --- | --- | --- | --- |
|  |  | Disagree/ Somewhat Disagree | Somewhat Disagree/ Somewhat Agree | Somewhat Agree/ Agree |  | Common Factor Reintegration to Normal Living |
| 1 |  | -1.558 | -1.136 | -0.636 |  | 0.793 |
| 2 |  | -1.309 | -0.799 | -0.220 |  | 0.875 |
| 3 |  | -0.724 | -0.349 | -0.040 |  | 0.888 |
| 4 |  | -1.902 | -1.235 | -0.595 |  | 0.768 |
| 5 |  | -1.098 | -0.629 | -0.054 |  | 0.787 |
| 6 |  | -0.888 | -0.349 | 0.288 |  | 0.796 |
| 7 |  | -1.189 | -0.550 | 0.035 |  | 0.846 |
| 8 |  | -1.455 | -0.999 | -0.328 |  | 0.898 |
| 9 |  | -1.795 | -1.325 | -0.707 |  | 0.777 |
| 10 |  | -2.066 | -1.161 | -0.479 |  | 0.547 |
| 11 |  | -1.670 | -0.990 | -0.117 |  | 0.784 |
| *Note:* RNLI = Return to Normal Living Index. Factor structure of Model 4 corresponds to the common factor structure proposed by Mothabeng et al. (2012). All factor loadings are significant with p < 0.001. | | | | | | |

**Supplementary Table 5**

*Correlations with 95% Confidence Intervals in the Two-Factor-Models of the RNLI for the Different Groups*

| **Model** | Group 1 (n = 153) | Group 2 (n = 74) |
| --- | --- | --- |
| Model 2 | 0.857 [0.762, 0.953] | 0.969 [0.891, 1.000] |
| Model 3 | 0.898 [0.837, 0.958] | 0.933 [0.854, 0.973] |
| *Note*. Model 2 corresponds to the structure proposed by Wood-Dauphinee et al. (1988), Hitzig et al. (2012, and Daneski et al. (2003). Model 3 corresponds to the structure proposed by Miller et al. (2011). Group 1= patients interviewed 6 months after sepsis, Group 2= informal caregiver or both interviewed 6 months after sepsis. RNLI = Return to Normal Living Index. All correlations are significant with p < 0.001. | | |

**Supplementary Figure 1**

*Patient Flow Chart*


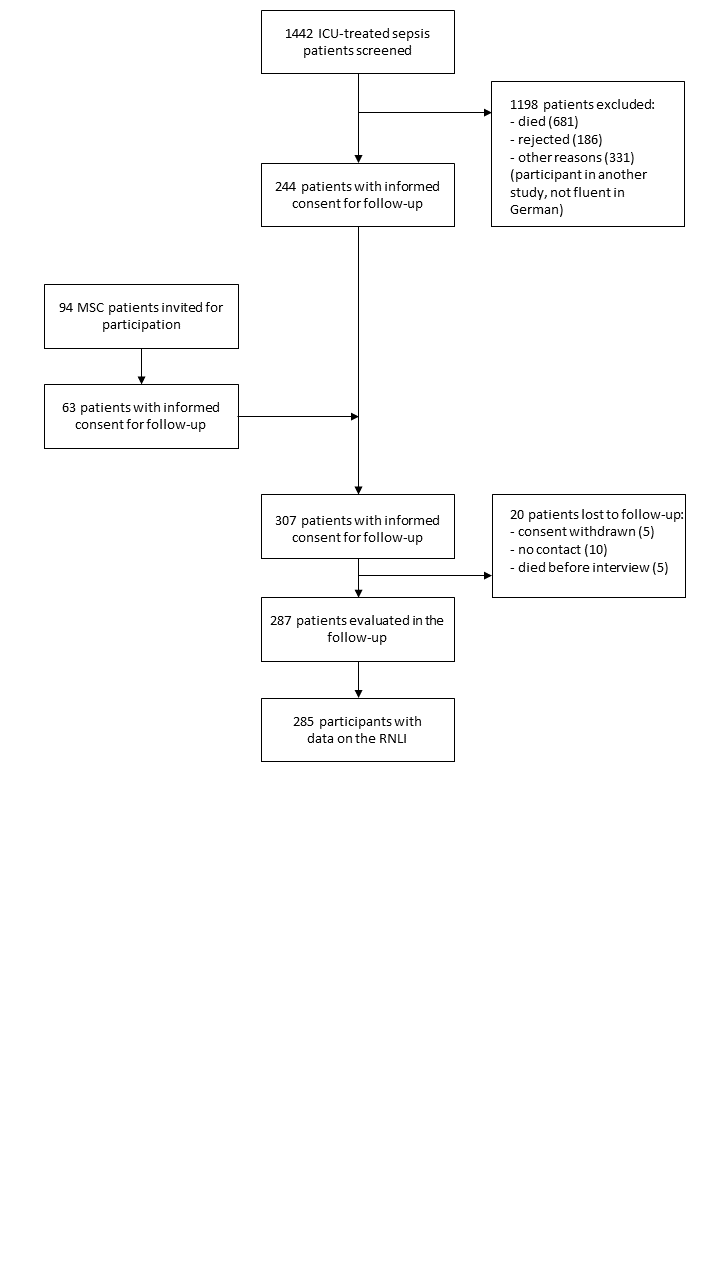


*Note*. ICU = intensive care unit, MSC = Mid-German Sepsis Cohort, RNLI = Return to Normal Living Index
